# Circulating MicroRNAs in Blood and Other Body Fluids as Biomarkers for Diagnosis, Prognosis, and Therapy Response in Lung Cancer

**DOI:** 10.3390/diagnostics11030421

**Published:** 2021-03-02

**Authors:** Luis Vicente Gayosso-Gómez, Blanca Ortiz-Quintero

**Affiliations:** Research Unit, Instituto Nacional de Enfermedades Respiratorias Ismael Cosío Villegas, Calzada de Tlalpan 4502, Colonia Sección XVI, Mexico City 14080, Mexico; lvg1_1@hotmail.com

**Keywords:** circulating microRNAs, lung cancer, non-invasive biomarkers, body fluids, diagnosis, prognosis, therapy response

## Abstract

The identification of circulating microRNAs (miRNAs) in peripheral blood and other body fluids has led to considerable research interest in investigating their potential clinical application as non-invasive biomarkers of cancer, including lung cancer, the deadliest malignancy worldwide. Several studies have found that alterations in the levels of miRNAs in circulation are able to discriminate lung cancer patients from healthy individuals (diagnosis) and are associated with patient outcome (prognosis) and treatment response (prediction). Increasing evidence indicates that circulating miRNAs may function as mediators of cell-to-cell communication, affecting biological processes associated with tumor initiation and progression. This review is focused on the most recent studies that provide evidence of the potential value of circulating miRNAs in blood and other body fluids as non-invasive biomarkers of lung cancer in terms of diagnosis, prognosis, and response to treatment. The status of their potential clinical application in lung cancer is also discussed, and relevant clinical trials were sought and are described. Because of the relevance of their biological characteristics and potential value as biomarkers, this review provides an overview of the canonical biogenesis, release mechanisms, and biological role of miRNAs in lung cancer.

## 1. Introduction

Lung cancer is the leading cause of death due to malignancy worldwide [1]. Its high mortality is mainly attributed to most patients being initially diagnosed in the late stages of the disease [2,3]. This late diagnosis is due to a combination of the absence of specific clinical symptoms in the early stages, a lack of effective screening methods, and confirmatory diagnosis being often postponed as it requires tissue samples to be obtained using invasive techniques [3]. Low-dose computed tomography (CT) is an available screening test recommended only for high-risk heavy smokers; because it may produce false-positive results or overdiagnosis, there is a potential risk of harm from repeated exposure to radiation, and its use is linked with increased healthcare-associated costs [4,5,6]. Importantly, the choice of therapy for lung cancer is based on the tumor histological subtype classification, stage of the disease, and presence of targetable mutations. The prognosis is highly dependent on the stage at initial diagnosis, which is predominantly poor due to late diagnosis [2,7]. Therefore, alternative biomarkers are urgently needed for the early and accurate diagnosis, classification, prognosis, and prediction of therapeutic response in lung cancer. Due to the risks associated with undergoing lung tissue biopsy or resection procedures, the use of novel biomarkers obtained from biological samples through minimally invasive or non-invasive methods is preferred, such as peripheral blood or other body fluids.

Concurrent with the discovery of cell-free or circulating microRNAs (miRNAs) in blood circulation in 2008, levels of these miRNAs in serum and plasma were found to be able to distinguish lung, prostate, and colorectal cancer patients from healthy individuals [8,9]. The high stability of circulating miRNAs in plasma and serum, their resistance to harsh storage conditions, and their quantifiability make them attractive targets for the development of cancer biomarkers [8,9]. In addition, circulating miRNAs are present in several other body fluids such as saliva, urine, pleural effusions, sputum, and bronchoalveolar lavage fluid, among others; similar to miRNAs in serum and plasma, alterations in their levels have been associated with diagnosis of cancers [10], including lung cancer [11,12,13]. Furthermore, it was discovered that cell-free miRNAs are released by tumor cells into the extracellular space and delivered into recipient cells in the tumor microenvironment, where they function as regulators of gene expression. As is the case with their counterpart, endogenous miRNAs, cell-free miRNAs have been associated with cancer-related processes such as metastasis, epithelial–mesenchymal transition (EMT), angiogenesis, and evasion of immune response, as part of a cell-to-cell communication mechanism in cancer [14].

Therefore, the potential role of circulating miRNAs in cancer-related processes and their stable and quantifiable form in peripheral blood and other body fluids emphasizes their potential clinical value as non-invasive biomarkers of lung cancer. This review summarizes current studies that provide evidence of the potential value of miRNAs in circulation and other body fluids as non-invasive biomarkers for lung cancer diagnosis, prognosis, and prediction of response to treatment.

## 2. miRNAs

**Figure 1:**
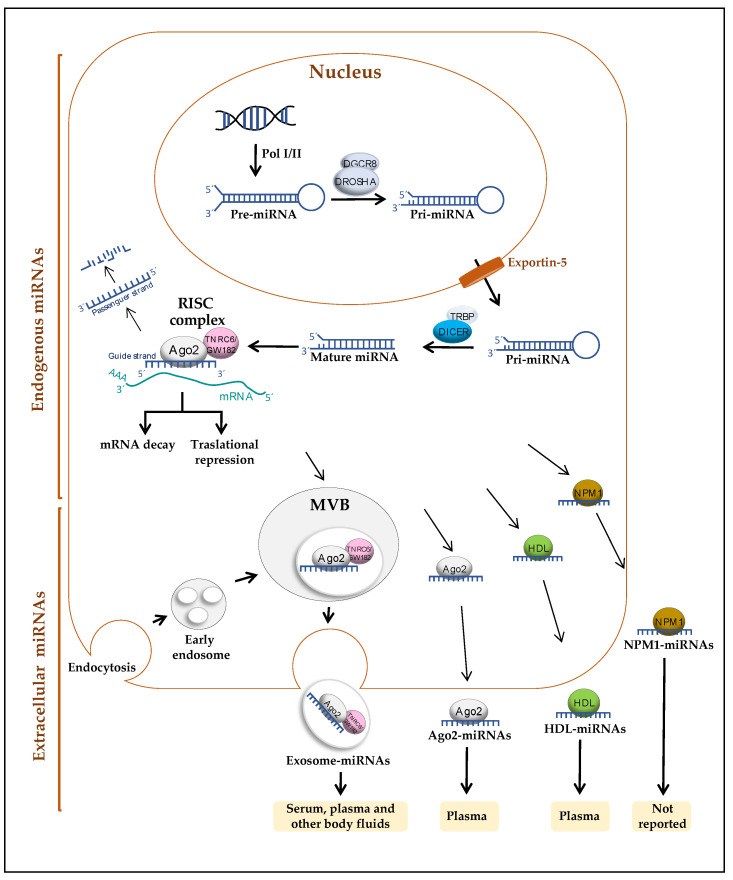
Schematic representation of the mechanisms for biogenesis and release of microRNAs (miRNAs) into extracellular space and body fluids.

miRNAs are small noncoding RNA molecules ~22 nucleotides (nt) in length that regulate the gene expression of several biological processes at the post-transcriptional level by blocking the translation of target messenger RNAs (mRNAs) [15]. In the canonical biogenesis pathway (Figure 1), which is the predominant pathway found in metazoans, miRNA genes are transcribed in the nucleus by RNA polymerase II (Pol II) to produce a primary precursor of ~60–100 nt called pri-miRNA [16]. Pri-miRNAs are cleaved by the Drosha/DGCR8 ribonuclease complex to produce a precursor pre-miRNA of ~70 nt [17], which is exported to the cytoplasm through the exportin-5/GTP-binding nuclear protein Ran (RanGTP) complex. Pre-miRNAs are further cleaved by the Dicer/TRBP complex (also known as Dicer/GW182) to finally produce the mature double-stranded miRNA of ~22 nt [18]. Mature miRNAs are loaded into the RNA-induced silencing complex (RISC) by one of its components, Argonaute 2 (AGO2) protein [19]. Then, the double strand dissociates, and only one is retained (guide strand), whereas the other is degraded (passenger strand); the guide strand binds to a partially complementary sequence in its target mRNA (in metazoans) [20,21], inducing the repression of gene expression through mechanisms of mRNA destabilization (also known as mRNA decay) and translational repression, although mRNA destabilization is the dominant effect where mammalian miRNAs are concerned [22,23].

Under normal physiological conditions, miRNAs regulate the gene expression of essential biological processes in virtually every cell and organism investigated to date, which include proliferation, differentiation, cell cycle, and apoptosis [24,25,26]. The expression of these endogenous miRNAs is also required for the proper development of several organs and systems such as the heart, central nervous system, and respiratory system, among others [27,28,29]. Under pathological conditions, endogenous miRNAs show altered expression patterns in cells and tissues that are associated with the presence of several cancers, including lung cancer [30,31]. These altered endogenous miRNAs can function as pro-oncogenic or anti-oncogenic regulators of cancer-related biological processes [32,33]. In lung cancer, several studies have provided evidence that endogenous miRNAs regulate cell invasion, migration, proliferation, epithelial–mesenchymal transition (EMT), metastasis, and therapy resistance [34,35,36,37].

### 2.1. Release Mechanisms of miRNAs

In 2007, miRNAs were reported to be released by cells into the extracellular space, and these cell-free miRNAs are transferable and functional in recipient cells [38,39]. Cell-free miRNAs can be released and uptaken by cells through vesicle trafficking and protein carrier mechanisms, and they are able to function as gene expression regulators in cell-to-cell communication mechanisms under normal and pathological conditions, such as cancer [10,14,38,39,40].

The known mechanisms for miRNA release include (1) release within extracellular vesicles (EVs), mainly exosomes [38]; (2) release of miRNAs associated with high-density lipoprotein (HDL) [41]; (3) release of miRNAs complexed with the protein Argonaute 2 (AGO2) [42]; and (4) release of miRNAs associated with the RNA-binding protein nucleophosmin (NPM1) [43] (Figure 1).

EVs is a generic term for particles naturally released from the cell that are delimited by a lipid bilayer and cannot replicate [44]. Exosomes are a type of small EVs, 40–150 nm in diameter, that are formed by the internal budding of endosomes to produce multivesicular bodies (MVBs). As cargo, exosomes transport miRNAs and other RNAs (mRNA and other noncoding RNAs), DNA, proteins, and lipids [45,46]. In the cytoplasm, miRNAs are loaded into MVBs by a process that requires neutral sphingomyelinase 2 (nSMase2), endosomal sorting complex transport machinery (ESCRT), sumoylated heterogeneous nuclear ribonucleoprotein A2B1 (hnRNPA2B1), and the AGO2 protein [47,48,49,50,51]. These MVBs fuse with the plasma membrane and are subsequently released as exosomes into the extracellular space [45]. In the extracellular space, exosomes can bind to the plasma membrane of recipient cells using several exosomal molecules, which include tetraspanins, heparan sulfate proteoglycans, and lectins [52,53,54]; consequently, exosomes are internalized by recipient cells via endocytosis, micropinocytosis, phagocytosis, or lipid rafts [54,55,56].

High-density lipoprotein (HDL) is one type of protein complex that transports fat molecules (lipids) though the body. HDL transports miRNAs in human plasma from healthy subjects and patients with atherosclerosis. In addition, HDL–miRNAs from the plasma of atherosclerotic patients were transferred into a cultured hepatocyte cell line, affecting target mRNAs. Complexes of HDL–miR-223 are also delivered into endothelial cells and suppress the expression of intercellular adhesion molecule 1 (ICAM-1) [41,57]. The mechanism of miRNA loading onto HDL in the cell is not completely understood, but HDL–miRNA binding could occur via divalent cation bridging [57].

Argonaute 2 (AGO2) is the main component of the RISC; it was found to be associated with miRNAs in human plasma and serum and to confer protection from RNase activity [42,58].

One study reported that following deprivation of serum in the culture medium, the human fibroblast HepG2 cell line releases exosomes-free miRNAs bound to the RNA-binding protein NPM1 [43].

Overall, the data indicate that miRNAs transported via exosomes and HDL are delivered into recipient cells in a functional way, where they target mRNAs and regulate gene expression, but there is no evidence of such a mechanism for miRNAs associated with AGO2 and NPM1 to date.

### 2.2. Circulating miRNAs in Blood and Other Body Fluids

In 2008, Mitchell et al. [9] found cell-free miRNAs in the plasma of healthy donors, while Chen et al. [8] confirmed their presence in the plasma and serum of healthy individuals. These cell-free or circulating miRNAs were found to be resistant to RNase digestion, several cycles of freezing and thawing, and extreme pH [8,9,58]. Since these initial studies, levels of circulating miRNAs in the plasma and serum of healthy individuals were found to be constant [8,59], whereas the levels in cancer patients (including lung cancer) are altered. These altered miRNAs could be used to distinguish cancer patients from healthy individuals, prompting the search for miRNAs that may be used as non-invasive biomarkers for diagnosis and prognosis [8,9,10,14].

Circulating miRNAs are present in other body fluids from healthy individuals, including tears, urine, amniotic fluid, colostrum, breast milk, bronchial lavage, cerebrospinal fluid, peritoneal fluid, pleural fluid, seminal fluid, saliva, and gastric juices [10]. Circulating miRNAs show a distinctive expression pattern in each body fluid, which can be used to distinguish the body fluid from which they originated. This distinctive expression pattern may suggest that miRNAs present a specific biological function associated with the surrounding tissues under normal physiological conditions [10,60,61]. Under pathological conditions, altered levels of miRNAs in body fluids have been associated with the diagnosis of several cancers, including bladder [62], renal [63], gastric [64], pancreatic [65], brain [66], and lung cancers [67,68].

## 3. Circulating miRNAs as Biomarkers of Cancer

Circulating miRNAs have several desirable characteristics that make them suitable as biomarkers for clinical applications: they are highly stable in biological samples, they can be obtained using relatively non-invasive isolation methods, they can be quantified using highly sensitive and accurate measurement methods (such as real-time quantitative polymerase chain reaction, or qPCR), and their levels seem to vary in the presence of a pathological process or disease.

In cancer, an ideal diagnostic biomarker must be associated with the presence of cancer cells or the malignant process; a prognostic biomarker, with the recurrence or progression of cancer or likelihood of a clinical event; and a response biomarker, with a biological response after treatment. In lung cancer, where the gold-standard method for diagnosis requires lung tissue samples, it is an additional advantage if the biomarker can be easily obtained on multiple occasions using non-invasive methods for monitoring of progression or therapy response. Therefore, circulating miRNAs have prompted a large number of research studies and economic investments toward exploring their potential clinical application as biomarkers of lung cancer.

Generally, the preclinical exploratory phase for diagnostic biomarker discovery consists of identifying circulating miRNAs whose expression levels, mainly in serum and plasma, are altered in cancer patients compared to healthy individuals. The second phase consists of a systematic evaluation of the ability of the candidate biomarker to distinguish subjects with cancer from individuals without cancer with high sensitivity and specificity and in a larger and independent study cohort. Lastly, additional studies are required to assess the accuracy of the previous results in the general population and in prospective large study cohorts.

**Figure 2:**
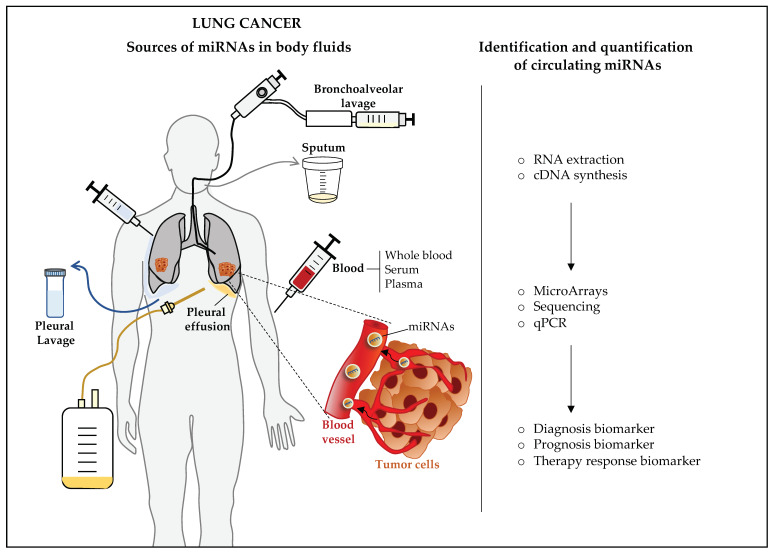
Sources of microRNAs (miRNAs) in body fluids for lung cancer biomarker discovery. miRNAs have been obtained from peripheral-blood-derived samples, bronchoalveolar lavage fluid, pleural effusion, pleural lavage fluid, and sputum of lung cancer patients. Available identification and quantification technologies include microarrays, next-generation sequencing, and real-time quantitative PCR (qPCR). Each technology requires the extraction of RNA from the biological fluid and the synthesis of complementary DNA (cDNA) by reverse transcription (RT).

Several technologies and platforms are available for the identification and quantification of circulating miRNAs in body fluids. The most used methods are next-generation sequencing (NGS), microarrays, and qPCR [69]. Each technology requires the extraction of RNA from the biological fluid and the subsequent synthesis of complementary DNA (cDNA) by reverse transcription (RT) (Figure 2). These technologies have differences in sensitivity, specificity, cost, and processing [69,70,71]. In general, NGS and microarrays have low sensitivity and specificity compared with available qPCR technologies, which have been shown to have high sensitivity and accuracy when miRNAs from serum and plasma are analyzed [72]. qPCR offers relatively simple and cost-efficient processing of samples and data. However, NGS and microarray platforms allow the simultaneous identification of multiple miRNAs (even hundreds of miRNAs) in a qualitative and semiquantitative approach; therefore, they are the methods of choice for performing wide screening. The use of different technologies and platforms may be one of the main reasons for the heterogenous results reported by various published studies regarding circulating miRNAs as biomarkers of cancer. Due to the above, the use of one standardized method is recommended. In addition, if semiquantitative sequencing or hybridization methods are used for miRNA discovery, qPCR should be used for the validation of findings to verify the quality and accuracy of the results.

The processing and quantification of circulating miRNAs face specific challenges related to their pre-analytical variables, which include the use of an efficient RNA extraction method, an efficient method for cDNA synthesis, verification of the sample quality (presence of hemolysis), verification of the RNA extraction quality using spike-in methods, and the use of proper normalizers [14,73,74]. Because miRNAs are present at very low levels in body fluids, small variations in these pre-analytical variables may significantly alter the analysis of circulating miRNA levels [14].

To verify that pre-analytical and analytical variables do not affect negatively affect the accuracy and consistency of results, the investigations must provide a complete and clear report of the methods and results sections. The report should include the experimental design, clinical data, size of the cohorts, sample collection and processing methods, and the analysis procedure.

An additional aspect of the experimental design that influences the reliability of data is the number of samples or participants included in the study. The variability between individuals regarding genetic and epidemiologic backgrounds may contribute to heterogeneous and inconsistent results regarding biomarker discovery; therefore, the findings should be validated in large and independent validation cohorts.

Cancer is a multifactorial disease that involves genetic, epigenetic, and environmental risk factors [75,76,77]; therefore, it is less probable that a single miRNA would be better than two or more candidate miRNAs as biomarkers for a diagnostic method with the sensitivity and specificity required for clinical application. Recent studies have shown that a combination of two or more miRNAs performs better than one single miRNA regarding specificity and sensitivity for diagnosis [78,79,80]. Accordingly, several studies have investigated signatures or panels of miRNAs (two or more miRNAs) to improve the sensitivity and specificity of the diagnostic detection compared with the use of a single candidate miRNA.

## 4. Circulating miRNAs as Biomarkers of Lung Cancer

Potential circulating miRNA biomarkers of lung cancer have been reported in serum, plasma, whole blood, bronchoalveolar lavage fluid, pleural effusion, pleural lavage fluid, and sputum (Figure 2); however, plasma and serum are the most extensively studied samples to date. Hypothetically, circulating miRNAs (mainly within exosomes) are released by lung tumor cells into the bloodstream and other body fluids, where they can be extracted, quantified, and potentially used as non-invasive biomarkers of the carcinogenesis process or the presence of cancer (Figure 2). On the other hand, the origin of miRNAs in the bloodstream could be attributed to platelets or other abundant hematopoietic cells in the blood, which indicates that the miRNA profile in circulation may also be a result of the physiological response to the presence of cancer. Nevertheless, a cancer biomarker should be associated with the presence of the tumor or the malignant process. Following this rationale, some studies have investigated the potential use of tumor-cell-derived miRNAs in circulation as cancer biomarkers [81,82]; alternatively, circulating miRNAs with altered levels in lung cancer patients have been further investigated for potential cancer-associated functions [83,84].

In the next section, we summarize the recently published studies that have reported on circulating miRNAs as biomarkers for the diagnosis, therapy response, and prognosis of lung cancer. We emphasize the studies that present desirable strengths in their research, such as clear and accurate reporting of their methods and results sections, the use of large-scale cohorts, and the analysis of a panel of miRNAs rather than a single miRNA.

### 4.1. Circulating miRNA as Diagnostic Biomarkers of Lung Cancer

#### 4.1.1. Whole Blood, Serum, and Plasma

Table 1 summarizes the circulating miRNA signatures reported by recent publications as potential diagnostic biomarkers of lung cancer in peripheral blood. In 2020, Fehlmann et al. [85] published a retrospective multicenter study with a large-scale cohort of 3066 subjects divided into four study groups, which included patients with (a) lung cancer (*n* = 606), (b) nontumor lung diseases (*n* = 593), (c) other diseases not affecting lungs (*n* = 883), and (d) unaffected control subjects (*n* = 964). The authors investigated the diagnostic value of various panels (or signatures) of miRNAs in whole-blood samples using miRNA microarrays to identify the candidate miRNAs. The results revealed a first signature of 15 miRNAs that distinguished patients with lung cancer from all other subjects in the study, with an area under the curve (AUC) of 0.965. A second signature of 14 miRNAs (AUC, 0.977) was able to distinguish patients with lung cancer from patients with nontumor lung diseases, which included mostly chronic obstructive pulmonary disorder (COPD). A third signature of 14 miRNAs (AUC, 0.960) distinguished early-stage patients with lung cancer from subjects without lung cancer. Patients in the group with diseases not affecting the lungs had multiple sclerosis, Parkinson’s disease, breast cancer, endometriosis, or various heart diseases; were undergoing abdominal surgery; or were developing sepsis. The strength of this study is the large-scale cohort and the inclusion of comparative study groups with nontumor lung diseases and other types of diseases that did not affect the lungs. Because of the size of the cohort, this study may be considered one of the most promising in the field, while the inclusion of comparative diseases in addition to the control group allowed analyzing the discriminative capacity of the discovered miRNAs for the specific diagnosis of lung cancer. However, this study did not apply a quantitative method, such as qPCR, to validate the findings that had been obtained using a microarray platform. Using whole blood may be also a disadvantage because the source of the miRNAs includes the endogenous miRNAs derived from the rupture of the blood cells (erythrocytes, platelets, and leucocytes), in addition to cell-free miRNAs in circulation. These different cells express specific sets of miRNAs, which may not be related to the presence of cancer, which would introduce variables that affect the levels of the miRNAs detected in the sample. However, using whole blood eliminates one laboratory procedure required for separation of serum or plasma and may simplify the analysis of miRNA biomarkers in clinical laboratory practice.

In 2020, Ying et al. [86] identified a five-miRNA signature in serum samples for the diagnosis of early-stage non-small-cell lung cancer (NSCLC) in a large cohort of 744 NSCLC cases (stages I and II) and 944 matched controls. The authors used qPCR for quantification of the miRNAs. They analyzed 540 miRNAs in serum samples from a discovery cohort consisting of 180 NSCLC patients and 216 control subjects (male Chinese healthy smokers). A panel of five miRNAs were selected (let-7a-5p, miR-1-3p, miR-1291, miR-214-3p, and miR-375) and tested in two independent verification cohorts consisting of 242 NSCLC patients and 190 controls (Chinese) and 101 NSCLC patients and 117 controls (Caucasians), which included men, women, smokers, and nonsmokers. This signature was tested in three additional cohorts consisted of 120 patients vs. 117 controls (Chinese), 67 patients vs. 273 controls (Chinese), and 34 patients vs. 31 controls (Chinese and Singaporean), which included stage II and IV cases. The results indicated that the five-miRNA signature was able to differentiate NSCLC cases from noncancer controls for all stages in all six study cohorts, as calculated from a logistic regression model. This signature showed a sensitivity of 81.3% (95% CI, 78.2–84.1%) for all cancer stages, a sensitivity of 82.9% (95% CI, 79.8–85.7%) for stages I and II, and a sensitivity of 83.0% (95% CI, 79.6–85.9%) for stage I NSCLC when the specificity was 90.7% (95% CI, 88.3–92.8%), which showed a potential diagnostic value for stage I and II NSCLC patients compared with matched controls regardless of gender and smoking status.

Concurrently, Asakura et al. [87] reported a two-miRNA signature in serum for the diagnosis of resectable lung cancer (miR-1268b and miR-6075), which was independent of histological type and stage. In this study, the discovery set included 208 lung cancer and 208 noncancer serum samples, and the validation set consisted of 1358 lung cancer and 1970 noncancer serum samples. The authors profiled the expression of 2588 miRNAs in serum samples using a microarray platform. The results revealed that one single miRNA (miR-17-3p; AUC, 0.935; sensitivity, 93.3%; specificity, 88.5%) was effective in distinguishing cancer patients, but a combination of two miRNAs (miR-1268b and miR-6075) improved the AUC relative to the single miRNA (diagnostic index = (−3.56049 × miR-1268b) + (1.99039 × miR-6075) + 16.7999; AUC, 0.993; sensitivity, 99.0%; specificity, 99.0%). The authors reported that the diagnostic index exhibited high performance for all pathological stages (IA, 96.1%; IB, 93.7%; IIA, 97.3%; IIB, 96.7%; IIIA, 90.2%; IIIB, 83.3%; IV, 100%) and histological types (adenocarcinoma, 95.1%; squamous cell carcinoma, 94.2%; small-cell lung cancer, 90.9%). In addition, the diagnostic indexes of miR-17-3p, miR-1268b, and miR-6075, individually, and of the two-miRNA panel (miR-1268b and miR-6075) significantly decreased after surgery, as determined in 180 lung cancer patients. This study is the largest regarding lung cancer samples and that reported a two-miRNA signature for the diagnosis of lung cancer. Importantly, the diminished diagnostic index after tumor resection supports its diagnostic value due to the presence of tumor tissue. However, the findings relied only on the results from a microarray platform and were not validated with a quantitative and more accurate method such as qPCR.

In 2018, Lu et al. [88] reported on a retrospective multicenter study aimed to identify plasma miRNAs for the diagnosis of lung cancer (LC) and for discrimination of small-cell lung cancer (SCLC) from non-small-cell lung cancer (NSCLC) in a total large cohort of 676 LC and 456 high-risk healthy subjects. They screened 723 microRNAs using a microarray platform with the plasma from 73 LC (52 NSCLC and 21 SCLC) and 33 high-risk healthy subjects; the results were further validated with qPCR in two independent validation cohorts of 345 LC (278 NSCLC and 67 SCLC) and 220 high-risk healthy subjects, and 258 LC (209 NSCLC and 49 SCLC) and 203 high-risk healthy subjects. A signature of six microRNAs (miR-17, miR-190b, miR-19a, miR-19b, miR-26b, and miR-375) discriminated lung cancer patients from healthy individuals (AUC, 0.868), while a signature of three microRNAs (miR-17, miR-190b, and miR-375) discriminated SCLC from NSCLC patients (AUC, 0.869). The advantages of this study are the large cohort, with samples collected from five medical centers, and use of a miRNA signature instead of a single miRNA as a potential diagnostic biomarker.

In 2020, Reiss et al. [83] investigated the diagnostic value of three signatures consisting of different combinations of several miRNAs in plasma for the early detection of lung cancer, but in a medium cohort. A total cohort of 139 samples consisting of 40 adenocarcinoma (AD), 38 lung squamous cell carcinoma (SCC), and 61 nondisease individuals were divided into a discovery cohort (38 patients and 21 controls) and a validation cohort (40 patients and 40 controls). This study analyzed 800 miRNAs in the discovery phase (using RNA sequencing technology) and validated the finding in the validation cohort using qPCR. The authors used three statistical methods to identify (a) an eight-miRNA signature consisting of miR-16-5p, miR-92a, miR-451a, miR-106b-5p, miR-155-5p, miR-217, miR-1285-3p, and miR-1285-5p (elastic net method); (b) a five-miRNA signature consisting of miR-16-5p, miR-148b-3p, miR-378e, miR-484, and miR-664a-3p (maximizing-R-square analysis/ or MARSA method); and (c) and a three-miRNA signature consisting of miR-16-5p, miR-92a, and miR-451a (C-statistic method). For the eight-miRNA signature, the specificity and sensitivity were 100% and 97%, respectively; for the five-miRNA signature, they were 97% and 100%, respectively; and for the three-miRNA signature, they were 100% and 84%, respectively. The authors chose the three miRNAs miR-16-5p, miR-92a, and miR-451a to perform bioinformatic analysis to identify molecular pathways regulated by those miRNAs. They reported that the three miRNAs are associated with several pathways related to tumorigenesis, such as rat sarcoma (Ras), mitogen-activated protein kinase (MAPK), and mammalian target of rapamycin (mTOR).signaling pathways. However, they did not provide experimental evidence of the association. The small size of the cohorts was the most relevant limitation; therefore, the findings will require further verification.

Pan et al. [89] also reported a two-miRNA signature for lung cancer diagnosis in a small cohort. They analyzed the levels of two previously identified miRNAs in lung cancer tissue (miR-33a-5p and miR-28-3p) in whole-blood samples from 90 patients with lung cancer and 90 healthy controls using qPCR. The combined diagnostic model showed that the AUC for the combination of two circulating miRNAs was 0.9511 (sensitivity, 96.67%; specificity, 83.33%). The authors further verified the diagnostic value of miR-33a-5p and miR-128-3p in 41 patients with lung cancer at an early stage (TNM stage I–II) and 41 healthy controls. The combined two-miRNA signature yielded higher AUC and specificity values than the individual miRNAs (AUC, 0.9554; sensitivity, 90.24%; specificity, 92.68%). The limitations of this study include the analysis of only two candidate miRNAs in the blood, the relatively small cohort size, and the clinical, epidemiological, and lung cancer histological type data not being provided for circulating miRNA investigation.

Other published studies have investigated circulating miRNAs transported within EVs—mainly exosomes—in serum and plasma as potential biomarkers of lung cancer. To analyze these exosomal miRNAs, it is necessary to first isolate the exosomes from liquid samples, mainly using ultracentrifugation methods that require special ultracentrifuge equipment and several hours for processing. In addition, exosome isolation from serum and plasma requires large volumes of these samples in comparison with the volume usually used for regular circulating miRNAs. These requirements may represent a disadvantage for future clinical use as biomarkers in a diagnostic test. However, the interest in exosomal miRNAs is based on evidence that has shown their role in carcinogenesis and tumor progression as regulators of cell-to-cell communication [14]. Hypothetically, cancer-related miRNAs are more likely to be suitable biomarkers for cancer diagnosis. Additionally, the function of the discovered miRNAs contained within exosomes can be further analyzed using in vitro and in vivo models [14]. One example of a study that investigated exosomal miRNAs as a potential diagnostic biomarker of lung cancer is by Jin et al. [81] in 2017. They aimed to identify tumor-derived exosomal miRNAs that discriminate lung adenocarcinoma (AD) from squamous cell carcinoma (SCC) in the early stages using plasma samples. Exosomes had previously been isolated using ultracentrifugation and immunoaffinity magnetic beads with anti-epithelial cell adhesion molecule (EpCAM), which was used as a marker for tumor-derived exosomes. Profiling of miRNAs from exosomes was performed in plasma of 26 stage I NSCLC patients (16 AD and 10 SCC) and 12 healthy controls using RNA sequencing technology. The findings were validated in a cohort consisting of 20 stage I NSCLC patients (10 AD and 10 SCC) and 30 healthy controls using qPCR. A final cohort of 42 NSCLC patients (31 AD and 11 SCC) was added to evaluate the findings amongst AD and SCC patients. The results showed that the levels of four miRNAs (miR-181b-5p, miR-361b-5p, miR-10b-5p, and miR-320b) discriminated NSCLC patients from non-NSCLC individuals (AUC, 0.899; sensitivity, 80.25%; and specificity, 92.31%); a signature of two miRNAs (miR-181-5p and miR-361-5p) discriminated AD from NSCLC patients (AUC, 0.936; sensitivity, 80.65%; and specificity, 91.67%); and a signature of two miRNAs (miR-320b and miR-10b-5p) discriminated SCC from NSCLC patients (AUC, 0.911; sensitivity, 83.33%; and specificity, 90.32%). The authors performed bioinformatic analysis to identify some predictive targets for the discovered exosomal miRNAs, such as insulin-like growth factor 1 (IGF1R), IGF2, mitogen-activated Protein kinase 8 (MAPK8), and serine/threonine-protein kinase 3 (AKT3). However, they did not provide further experimental evidence for the predictive findings. The limitations of this study that may prevent their clinical application include the complicated method used for the isolation of exosomes and the large volume of sample required (2.5 mL plasma). The size of the total cohort was small (less than 100 NSCLC samples); therefore, further analysis should be performed in a larger cohort to obtain sound evidence regarding the potential use of the exosomal miRNAs as diagnostic biomarkers. Another example is Zhan et al. [90] in 2020. They reported the analysis of exosomal miRNAs isolated from the serum of 330 NSCL patients and 312 healthy controls using microarray technology and qPCR. However, the miRNA profiling was performed in samples from only two NSCLC patients and one healthy control, which it is not considered a representative sample size. Six of twenty-two miRNAs that were found differentially expressed in NSCLC patients compared with healthy controls were further tested by qPCR, and the results indicated that miR-5684 and miR-125b-5p were downregulated in NSCLC patients. Diagnostic performance yielded an AUC of 0.793, a sensitivity of 82.7%, and a specificity of 62.1%. Zhang et al [91], also in 2020, evaluated the diagnostic value of exosomal miRNAs in the serum of early-stage NSCLC patients compared with healthy controls by microarray technology and qPCR. The authors performed miRNA profiling using one pool of healthy controls (consisted of five serum samples) and two pools of NSCLC patients (consisted of five non-metastatic NSCLC patients and five metastatic NSCL patients). They further evaluated 28 candidate miRNAs in 48 NSCLC patients and 48 healthy controls by qPCR. The results were validated in serum samples from two additional independent cohorts consisting of 72 NSCLC patients and 72 healthy controls, and 156 NSCLC patients and 162 healthy controls, respectively. They found that miR-20b-5p and miR-3187-5p were downregulated in NSCLC patients and the combination of the two miRNAs yielded an AUC of 0.848. Moreover, the diagnosis performance was improved by using carcinoembryonic antigen (CEA) in combination with miR-20b-5p and miR-3187-5p (AUC, 0.905), whereas the combination of miR-20b-5p, miR-3187-5p, and CEA for diagnosis of early-stage NSCLC (0 and I stages) showed an AUC of 0.930.

#### 4.1.2. Other Body Fluids

Table 2 summarizes the circulating miRNAs reported by recent publications as potential diagnostic biomarkers of lung cancer in other body fluids. Bronchoalveolar lavage (BAL) fluid is one source of miRNAs for lung cancer diagnosis, although there are few recent related published studies available. Another characteristic of these studies is the small size of the analyzed cohorts. One example is Kim et al. [67], published in 2018, who investigated the diagnostic value of six exosomal miRNAs (miR-7, miR-21, miR-126, let-7a, miR-17, and miR-19) isolated from BAL fluid for the diagnosis of early-stage lung adenocarcinoma. The authors reported that these six miRNAs were chosen based on previous reports that showed evidence of their diagnostic value for lung adenocarcinoma; however, they referred to a review-type paper [92] instead of a specific study or studies that provided such evidence. The study evaluated the six miRNAs in prospective BAL samples from 13 lung adenocarcinoma patients (stage I or II) and 15 control patients with nontumor pathology consisting of nonspecific interstitial pneumonia (*n* = 4), chronic eosinophilic pneumonia (*n* = 3), sarcoidosis (*n* = 2), cryptogenic organizing pneumonia (*n* = 2), hypersensitivity pneumonitis (*n* = 2), idiopathic pulmonary fibrosis (*n* = 1), and acute eosinophilic pneumonia (*n* = 1). The results showed that exosomal miRNA-126 and let-7a levels were significantly higher in the BAL fluid of lung adenocarcinoma patients than in control subjects. Regarding limitations, this study analyzed only six selected miRNAs (without proper explanation of the selection rationale) in a very small cohort of lung adenocarcinoma and control group subjects. The authors reported that they validated the results in four paired tumor and nontumor tissue samples, which is not an actual validation because, first, the number of samples was only four and, second, validation should be performed in the same type of sample (BAL fluid).

Another example is Rehbein et al. [93], who, in 2015, analyzed the circulating miRNAs in bronchial lavage (BL) fluid in a discovery cohort of 10 advanced lung cancer patients and 10 noncancerous individuals by PCR array and validated the results in an independent validation cohort of 30 lung cancer patients and 30 noncancerous subjects by qPCR. Lung cancer patients were diagnosed with squamous cell lung cancer (SCLC) or adenocarcinoma in an advanced stage (III-IV), and the noncancerous group with benign lung diseases. The authors reported a panel of five microRNAs (U6 snRNA, miR-1285, miR-1303, miR-29a-5p, and miR-650) that were significantly upregulated in patients with lung cancer compared to the noncancerous control group, although small nuclear RNA U6 is a different class of noncoding RNA to the miRNAs. The cohort size was very small; therefore, the finding should be validated in a larger cohort.

Malignant pleural effusion commonly occurs in the advanced stage of NSCLC, particularly in adenocarcinoma, due to tumor growth in the periphery of the lung, which may invade the pleural cavity. Some studies have investigated the potential diagnostic value of cell-free miRNAs in this body fluid for lung cancer. For example, in 2013, Han et al. [13] analyzed the miRNAs in pleural effusion samples in a discovery cohort of 10 patients with malignant benign pleural effusion (MPE) diagnosed with adenocarcinoma and 10 patients with benign pleural effusion (BPE) using microarray technology. The findings were validated in a cohort of 42 patients with MPE and 45 patients with BPE using qPCR. Additionally, the levels of cytokeratin 19 fragment (CYFRA 21-1) and carcinoembryonic antigen (CEA) were measured in the pleural effusions. The results showed that the diagnostic performance of miR-198 (AUC, 0.887; sensitivity, 71.1%; specificity, 95.2%) was comparable to that of the CEA (AUC, 0.898, sensitivity, 78.4%; specificity, 97.5%) and better than that of CYFRA 21-1 (AUC, 0.836; sensitivity, 78.4%; specificity, 82.5%). The combined use of the three markers yielded better performance with an AUC of 0.926, a sensitivity of 89.2%, and a specificity of 85.0%.

Most recently, the diagnostic value of circulating miRNAs has been analyzed in the liquid phase of pleural lavage of lung cancer patients. Pleural lavage cytology (PLC) is a technique for detecting subclinical dissemination of malignant cells in the pleural cavity; it is performed by injecting normal saline into the pleural space and aspirating at the time of thoracoscopy. In 2020, Watabe et al. [68] analyzed the clinicopathological significance of the expression levels of only one miRNA (miR-21) in extracellular vesicles (EVs) extracted from the pleural lavage fluid of 41 lung adenocarcinoma patients using digital PCR. They found that EV miR-21 levels in pleural lavage fluid are associated with positive cytology and pleural invasion in the primary sites, even in cytology-negative cases. In 2019, Roman-Canal et al. [12] analyzed the diagnostic value of EV miRNAs isolated from the pleural lavage fluid of 21 patients with adenocarcinoma (AD) and squamous cell carcinoma (SCC) and 25 control patients with benign pleural effusions using PCR array technology. Three individual miRNAs (miR-1-3p, miR-144-5p, and miR-150-5p) yielded the best accuracy for lung cancer diagnosis compared to controls. Both studies had small cohorts; therefore, further analysis is needed.

Sputum is another source of miRNAs in body fluids for lung cancer diagnosis; however, the actual source of miRNAs is the cell pellet collected from the sputum by centrifugation; therefore, they are considered to be endogenous rather than circulating or cell-free miRNAs. In 2014, Shen et al. [11] investigated miRNAs as potential biomarkers in the sputum of lung cancer patients to improve the specificity of computed tomography (CT). This study used qPCR and focused on 12 miRNAs that had been previously identified in lung tumor tissue. The training cohort included 66 lung cancer patients (13 SCLC and 53 NSCLC) and 68 cancer-free smokers (39 with chronic obstructive pulmonary disease (COPD), 16 with pneumonia, 7 with sarcoidosis, and 4 with inflammatory granuloma). The testing cohort included 64 lung cancer patients (6 SCLC and 58 NSCLC) and 73 cancer-free smokers (40 with COPD, 18 with pneumonia, 8 with sarcoidosis, and 7 with inflammatory granuloma). The results showed 10 differentially expressed miRNAs in lung cancer patients compared to controls, but the combination of two miRNAs (miR-31 and miR-210) showed better performance (AUC, 0.83) than any of the 10 miRNAs used alone or the 10 together. The use of the two miRNAs (miR-31 and miR-210) in combination yielded 65.2% sensitivity and 89.7% specificity, whereas CT had 93.9% sensitivity and 83.8% specificity for lung cancer diagnosis. The combined analysis of the two-panel miRNAs and CT showed a higher specificity than CT used alone (91.2%). Therefore, the authors suggested that this sputum two-miRNA biomarker might improve the performance of CT for diagnosis of lung cancer. Note that this study included high-risk smokers with unrelated cancer diseases as controls precisely to fulfill the main objective of analyzing markers that improve the specificity of the CT screening method used for lung cancer diagnosis. The authors acknowledged the need to perform a prospective study with a larger cohort to test the diagnostic use of this panel of miRNAs.

There are a few recent published studies regarding using sputum as the sample source. For example, in 2020, Liao et al. [94] (from the same research group as Shen et al. [11]) focused on the two miRNAs in sputum (miR-31 and miR-210) and three miRNAs in plasma (miRs-21-5p, 210-3p, and 486-5p) that they had previously identified [11,95]. Instead of testing their previous findings, the authors used qPCR to evaluate the combined use of the sputum and plasma miRNAs in a new training cohort of 76 NSCLC patients and 72 cancer-free smokers and a test cohort of 56 NSCLC patients and 55 cancer-free smokers. The authors reported that the panel consisting of the two sputum miRNAs (miRs-31-5p and 210-3p) and one plasma miRNA (miR-21-5p) had higher sensitivity (85.5%) and specificity (91.7%) for diagnosis of NSCLC compared with the individual miRNAs. Notably, the sensitivity and specificity were higher than those exhibited by the two-panel miRNAs in sputum (65% sensitivity and 89% specificity) [11] and the three-panel miRNAs in plasma (75% sensitivity and 85% specificity) reported in previous studies [95]. Contrary to the previously mentioned investigation, this study did not disclose the diseases non-related to cancer in the control groups, named cancer-free smokers [11], and verification of the adequacy of the normalizer used for qPCR was not reported. The cohort size was limiting, and the results will require further verification using a larger cohort.

### 4.2. Circulating miRNA as Therapy Response Biomarkers of Lung Cancer

Circulating miRNAs are also potential biomarkers of therapy response in lung cancer. The identification of patients with a positive response to a specific lung cancer treatment is essential to avoid unnecessary costs and potentially serious side effects for patients who are not expected to gain benefit from the treatment.

In 2020, Shukuya et al. [96] investigated the potential use of free circulating miRNAs and miRNAs within EVs in the plasma of patients prior to treatment as biomarkers for the anti–programmed cell death protein 1/programmed cell death protein 1 ligand (PD-1/PD-L1) antibody therapy response in advanced NSCLC. The study included a screening cohort of 29 pre-treated NSCLC patients, which consisted of 14 responders and 15 non-responders, and a validation cohort of 21 pre-treated NSCLC patients consisting of 8 responders and 13 non-responders. Patients who showed a partial response (PR) or stable disease (SD) lasting more than 6 months were classified as responders, and patients who showed progressive disease (PD) were classified as non-responders. From the screening cohort, 32 and 7 circulating miRNAs in plasma and EVs, respectively, were identified as differentially expressed between responders and non-responders using next-generation sequencing. Ten circulating miRNAs were chosen for qPCR validation using cohort 1, and nine were differentially expressed between responders and non-responders. Five were chosen for further validation in cohort 2 (miR-199a-3p, miR-200c-3p, miR-21-5p, miR-28-5p, and miR-30e-3p), and only three of these were significantly different (miR-200c-3p, miR-21-5p, and miR-28-5p) between responders and non-responders. Without providing a clear explanation, the authors included miR-199a-3p when analyzing the diagnostic value of the combination of two and three miRNAs; they reported that the highest-performing set was a combination of three miRNAs (miR-199a-3p, miR-21-5p, and miR-28-5p), which produced an AUC of 0.925. The authors reported that they could not validate the EV miRNAs by qPCR due, in part, to their low concentration, probably because they used only 200 μL of plasma and a size-exclusion column for EV purification, whereas most studies use 1–5 mL of the liquid sample for exosome isolation and ultracentrifugation methods. The analysis of circulating miRNAs as potential biomarkers for discrimination of responders from non-responders to anti-PD-1/PD-L1 antibody therapy is relevant due to the lack of reliable predictive markers in the clinic. However, the cohort size was small; therefore, the findings will require further analysis in a larger number of patients. Based on their findings, circulating miRNAs, and not EV miRNAs, should be further tested as potential biomarkers.

In 2020, Peng et al. [97] investigated the potential use of exosomal miRNAs from the plasma of patients with advanced epidermal growth factor receptor/anaplastic lymphoma kinase (EGFR/ALK)-negative NSCLC that received PD-1/PD-L1 inhibitors as biomarkers for the identification of patients with a positive response to immunotherapy. Five patients with a partial response (PR) to treatment were included as responders, and four patients with progressive disease (PD) were included as non-responders. Plasma samples from seven healthy individuals were included as normal controls. The nine patients were treated with different immunotherapy drugs targeting PD-1/PD-L1, and plasma samples were collected before treatment and after if the patients achieved a PR. One milliliter of plasma was used for exosome isolation by ultracentrifugation, and exosome-derived miRNAs were profiled using next-generation sequencing of small RNAs. The results showed that PD patients had higher levels of hsa-miR-320d, hsa-miR-320c, and hsa-miR-320b compared to PR patients, which might indicate correlation with an unfavorable response to anti-PD-1 treatment. Additionally, the authors reported that miR-125b-5p was downregulated in the post-treatment paired samples compared to the pre-treatment samples of the four PR patients; they suggested that it might be a potential indicator to monitor the efficacy of anti-PD-1 treatment. They also suggested that miR-125b-5p may act as a T cell suppressor based on an unrelated published study, but experimental or predicted evidence was not provided. This study had clear limitations: The first is the small number of samples (a total of nine, five responders and four non-responders), which greatly limited the reliability of the conclusions, which was acknowledged by the authors; this cohort size is too small, so epidemiological and clinical variables may have importantly affected the results (such as smoking status, NSCLC histological subtype, different immunotherapy drugs used, etc.). In addition, the findings relied only on the identification of miRNAs by sequencing and differential expression analysis, which should be validated using a more accurate and quantitative method such as qPCR.

In 2020, Fan et al. [98] analyzed serum miRNAs as potential biomarkers to predict the response to anti-PD1 therapy in advanced NSCLC patients. The study included 80 stage IV NSCLC patients divided into a discovery set consisting of 27 non-responders and 19 responders and a validation set of 17 non-responders and 17 responders. Patients with a partial or complete response to therapy were classified as responders. PCR array technology was used for miRNA profiling and individual qPCR for validation of the results. The results showed that miR-93, miR-138-5p, miR-200, miR-27a, miR-424, miR-34a, miR-28, miR-106b, miR-193a-3p, and miR-181a were significantly higher in non-responders than in responders (AUC, 0.975; 95% CI, 0.875–1.108; *p* < 0.0001).

Hojbjerg et al. [99] investigated the predictive value of four miRNAs (miR-30b, miR-30c, miR-221, and miR-222) in the plasma of epidermal growth factor receptor (EGFR)-mutated NSCLC patients receiving erlotinib. The study focused on these four miRNAs because they are associated with gefitinib resistance in lung cancer cell lines that harbor activating EGFR mutations [100]. The study included 29 NSCLC patients receiving erlotinib (7 with no progression and 22 with progression), with stage IV adenocarcinoma, with an activating mutation in the EGFR. Progression was defined as radiological progression judged by the response evaluation criteria in solid tumors (RECIST). Responders were defined as having a partial response, complete response, or clinical benefit for >6 months. Plasma levels of the four miRNAs (prior to treatment) were analyzed by qPCR. The results showed that low concentrations of miR-30b and miR-30c were significantly correlated with longer progression-free survival (PFS) (miR-30b: hazard ratio (HR) = 0.303 (0.123–0.747), *p* < 0.05; miR-30c: HR = 0.264 (0.103–0.674), *p* < 0.05), while low concentrations of miR-30c were significantly correlated with superior overall survival (OS; HR = 0.30 (0.094–0.954), *p* < 0.041). Therefore, the authors suggested that patients with high plasma concentrations of miR-30b and miR-30c are more likely to experience a reduced effect of erlotinib despite having an activating EGFR mutation. Due to the small sample size in this study, the finding should be confirmed in a larger cohort.

Table 3 summarizes the circulating miRNAs (signatures) reported by recent publications as potential therapy response biomarkers of lung cancer.

### 4.3. Circulating miRNA as Prognosis Biomarkers of Lung Cancer

A prognostic biomarker is used to identify the likelihood of a clinical event or recurrence or progression of cancer. Some studies have investigated the potential value of circulating miRNAs as prognostic biomarkers in the peripheral blood of lung cancer patients.

In 2020, Khandelwal et al. [101] used qPCR to investigate the levels of circulating miR-590-5p in the plasma of 80 patients diagnosed with NSCLC and 80 healthy individuals as controls. This miRNA was selected based on published evidence of its levels being dysregulated in several other cancers and its association with oncogenic and tumor-suppressive properties. The authors further evaluated the association of miRNA-590-5p expression with prognosis of NSCLC patients using Kaplan–Meier (K-M) and log-rank analyses. The results showed that miRNA-590-5p levels were downregulated in the plasma of NSCLC patients compared to controls. In addition, patients with low levels of miRNA-590-5p had significantly lower median survival rates in comparison to patients expressing high miR-590-5p levels (*p* < 0.0001; 95% CI, 1.412–3.498), suggesting that miR-590-5p is a potential prognostic marker for the progression of NSCLC.

In 2018, Xu et al. [102] analyzed the predictive value of 13 pro-angiogenic miRNAs for disease-free survival (DFS) and overall survival (OS) in the plasma of 196 NSCLC patients using qPCR. The median follow-up period was 56.7 months. The results of the K-M curves showed that high expression levels of miR-18a (*p* < 0.001), miR-20a (*p* < 0.001), miR-92a (*p* < 0.001), miR-126 (*p* < 0.001), miR-210 (*p* = 0.003), and miR-19a (*p* = 0.027) correlated with a worse DFS. In addition, NSCLC patients with high levels of miR-18a, miR-20a, miR-92a, miR-210, and miR-126 had a shorter OS than patients with low expression levels of these miRNAs (all *p* ≤ 0.001). The results suggested that plasma miR-18a, miR-20a, and miR-92a are potential biomarkers for prognosis of NSCLC patients.

In 2017, Zhao et al. [103] analyzed the association of plasma miR-34a/b/c expressions with clinicopathological properties and prognosis of NSCLC patients. They selected only miR-34 because of its altered expression reported in some cancers, including lung cancer, in addition to its association with prognosis of SCLC patients. The cohort consisted of 196 NSCLC patients, and the levels of plasma miR-34a/b/c were measured by qPCR. The results showed that elevated levels of miR-34a correlated with a prolonged DFS (*p* = 0.011) and OS (*p* = 0.011) compared to low levels of expression, whereas high levels of plasma miR-34c predicted a longer DFS (*p* = 0.038) than low levels of expression. The findings suggested that circulating miR-34a and miR-34c have potential prognostic value for NSCLC patients.

These three studies analyzed medium-sized cohorts but tested only one or selected miRNAs (angiogenic); therefore, the analysis was restricted to a limited number of miRNAs lacking previous screening or discovery investigation in plasma samples of NSCLC patients.

In 2018, Halvorsena et al. [104] investigated the predictive value of circulating microRNAs associated with overall survival after nivolumab treatment in the serum of NSCLC patients. Differently from the previous studies, the authors performed wide miRNA profiling using next-generation sequencing with the serum of 20 NSCLC patients prior to treatment with nivolumab (therapy of antibodies against the PD-1 receptor), followed by validation of the findings in a cohort of 31 patients using qPCR. A signature of seven miRNAs (miR-215-5p, miR-411-3p, miR-493-5p, miR-494-3p, miR-495-3p, miR-548j-5p, and miR-93-3p) was significantly associated with OS > 6 months (*p* = 0.001) with a sensitivity and specificity of 71% and 90%, respectively. Because the size of the cohort was relatively small, further validation in an independent and larger cohort is required.

Table 4 summarizes the circulating miRNAs (signatures) reported by recent publications as potential prognosis biomarkers of lung cancer.

## 5. Biological Role of Circulating miRNAs in Lung Cancer

Several studies have proposed that extracellular miRNAs, mainly via exosomes, are mediators of cell-to-cell communication under normal and pathological conditions. In addition, extracellular miRNAs can be transported via blood circulation or other body fluids and reach distant cells and tissue, where they function as gene expression regulators. There is evidence of the latter under normal physiological conditions; for example, adipose tissue releases exosomal miRNAs into circulation, which can reach distant liver tissue cells and regulate their gene expression [40]. Exosomal miRNAs also participate in cell-to-cell crosstalk between endothelial cells and smooth muscle cells, induced by atheroprotective stimuli [105].

In cancer, extracellular miRNAs are released by tumor cells into the tumor microenvironment, targeting resident or infiltrated normal cells or other tumor cells, affecting oncogenesis and tumor-progression-related mechanisms [106,107,108,109,110]. Several studies have reported that tumor-derived miRNAs, within EVs or exosomes, are present in the blood circulation and body fluids in several types of cancer. However, evidence of the regulatory effect of these miRNAs on cells and tissue that are distant or far from the site of origin of the tumor has not yet been reported. Nevertheless, potential tumor-derived miRNAs in circulation and body fluids can be extracted, quantified, and used as potential non-invasive biomarkers of cancer presence.

In lung cancer, exosomal miRNAs released by tumor cells into the tumor microenvironment have been associated with the regulation of proliferation, invasion, migration, EMT, angiogenesis, metastasis, and therapy resistance, indicating their role in lung carcinogenesis and tumor progression. For example, Wei et al. [82] found that the lung cancer cell line A549 releases exosomes enriched with miR-222-3p, which can be transferred to recipient lung cancer cells and enhance their proliferation, migration, invasion, and gemcitabine resistance, thereby promoting malignant phenotype characteristics and therapy resistance of lung cancer cells. Fan et al. [111] reported that overexpressed miR-210 transported within exosomes derived from lung cancer cells induces the transformation of recipient fibroblasts into cancer-associated fibroblasts (CAFs), which, in turn, produce proangiogenic factors such as vascular endothelial factor (VEGF). Lawson et al. [112] showed that miR-142-3p is enriched in EVs derived from lung cancer cells and is transferred to recipient fibroblasts and endothelial cells. Transfer of miR-142-3p into endothelial cells promotes angiogenesis though inhibition of transforming growth factor beta 1 receptor (TGFβR1), while transfer to fibroblasts induces transformation to a CAF phenotype: both changes are associated with tumor progression. He et al. [113] reported that miR-499-5p is overexpressed in exosomes derived from highly metastatic lung cancer cells, which can be transferred to recipient lung cancer cells, enhancing cell proliferation, migration, and EMT, which consequently contributes to cancer metastasis.

Hypothetically, circulating miRNAs potentially associated with carcinogenesis and tumor progression mechanisms in lung cancer may be relevant for biomarker discovery investigations because this association may improve the odds that they will have clinical value as biomarkers. However, circulating miRNAs have multiple cellular origins, which influence the expression profile and levels of miRNAs detected in liquid biological samples.

## 6. Clinical Application of Circulating miRNAs as Biomarkers of Lung Cancer

The goal of identifying clinically applicable circulating miRNAs as biomarkers of lung cancer seems far from being achieved. Despite the high number of published studies, since the discovery of circulating miRNA as potential biomarkers, the findings have been inconsistent or heterogeneous among studies. However, in recent years, the experimental strategy and techniques for miRNA identification and quantification have improved considerably, and standardized methods for reporting have been widely recommended, with some studies evaluating large cohorts and delivering promising results for lung cancer diagnosis. Nevertheless, the high number of published studies is inconsistent with the low number of clinical trials evaluating miRNAs as biomarkers in lung cancer. Currently, seven clinical trials are found registered in the US National Institutes of Health database ClinicalTrials.gov when using the keywords “miRNA and lung cancer”. Of these seven studies, three have passed their completion date and their status has not been verified in more than two years. From the remaining four clinical trials, one named Quantification of MicroRNAs in Diagnosis of Pulmonary Nodules (miR-Nod) aimed to analyze 34 miRNAs in the blood of patients with pulmonary nodules visible in a scanner (defined as a rounded picture of greater than 5 mm and less than 30 mm in the lung parenchyma) in a time frame of 18 months. The study description did not include any references to the preclinical analysis or the 34 miRNAs that will be tested. This clinical trial is reported as completed and included a medium cohort of 103 participants, with the completion date reported as May 2019. However, no study results have been posted on ClinicalTrials.gov, and we could not find any publication associated with this clinical trial.

Another study entitled Addition of MicroRNA Blood Test to Lung Cancer Screening Low Dose CT is a prospective observational, longitudinal, diagnostic study aiming to demonstrate the specificity of the Hummingbird Diagnostics microRNA test for lung cancer diagnosis (a company based in Heidelberg, Germany) in the total blood from a cohort of 479 participants undergoing lung cancer screening with low-dose computed tomography (LDCT) in the United States. This is an active clinical trial that is not currently recruiting participants. The description of this study informs that a “novel cancer test relying on microRNA signatures will be evaluated” in blood samples collected prior to the LDCT scan, and the results will be compared with the CT scan results and follow-up test for cancer diagnosis. The study description did not include any references to the preclinical analysis or miRNA signatures that will be tested. The estimated study completion date was posted as 30 August 2020; however, no study results have been posted on ClinicalTrials.gov, and we could not find any publication associated with this study. The results will likely be available in 2021.

Another study entitled Plasma MicroRNA Profiling as First Line Screening Test for Lung Cancer Detection: A Prospective Study (BIOMILD) is a large prospective study that aims to evaluate a panel of 24 miRNAs in the plasma of healthy high-risk heavy smokers as biomarkers for early diagnosis of lung cancer. This is an active clinical trial that is not currently recruiting participants. The actual enrollment reported for this large study was 4119 participants (healthy heavy smokers), and the estimated date of completion was posted as September 2021. The panel of miRNAs was based on the results from three previous studies referred to in the description of the clinical trial [31,114,115]. The miRNA signature will be evaluated, and the risk of lung cancer will be determined through a program of active surveillance based on the calculated miRNA risk profile regarding the development of lung cancer and the aggressive form of the disease over a time frame of three years. All participants will undergo baseline LDCT, spirometry, and miRNA profiling. Participants with a low-risk miRNA profile will repeat the miRNA assay and LDCT at 2 years, while participants with a high-risk miRNA profile will undergo additional diagnostic tests consisting of positron emission tomography (PET) in the case of suspicious CT or positive computed tomography (CT) or needle aspiration biopsy in the case of negative CT. This is one of the largest studies, so far, for lung cancer diagnosis in its clinical phase, but we will have to wait for the outcome, probably at the end of 2021 or early 2022.

The last clinical trial registered on ClinicalTrials.gov is an active study still recruiting participants, titled Optimized Lung Cancer Screening to Prevent Cardiovascular and Pulmonary Diseases Coupled with Primary Prevention (SMAC-1). This prospective study aims to validate circulating markers to enhance LDCT sensitivity and specificity for the screening of lung cancer, cardiovascular disease (CVD), and chronic obstructive pulmonary disease (COPD) in a high-risk population. The time frame of this study is planned from 10 October 2018 to 10 October 2021, and the estimated enrollment is 2000 participants. Among the different circulating biomarkers that will be tested, a signature of six miRNAs will be evaluated; however, this panel of miRNAs is associated with the increase or reduction in the risk of CVD major adverse cardiovascular events (MACE) in a previous study (the reference is not provided). Therefore, the evaluation of these six selected miRNAs is not aimed at investigating the diagnostic value for lung cancer. Table 5 summarizes the six clinical trials that investigated the value of circulating miRNAs as biomarkers of lung cancer that are registered on ClinicalTrials.gov.

In conclusion, there is currently one promising study with a large cohort for the evaluation of circulating miRNAs in the early diagnosis of lung cancer (Plasma MicroRNA Profiling as First Line Screening Test for Lung Cancer Detection), the results of which will probably be available in late 2021 or early 2022. We should also search for results from the study titled Addition of MicroRNA Blood Test to Lung Cancer Screening Low Dose CT that tested the Hummingbird Diagnostics microRNA test for lung cancer diagnosis, which may also be relevant for the field and hopefully will be made available in 2021.

## 7. Conclusions

The biological characteristics of circulating miRNAs in blood and other body fluids as well as their role in cancer-related mechanisms highlight their great potential as non-invasive biomarkers of lung cancer. However, in spite of the high number of published studies and the efforts directed toward clinical application, this goal seems far from being achieved. Several reasons for this have been discussed, among them being the small size of cohorts used in analysis, the use of non-standardized methods for quantification of circulating miRNAs, and the small concentration of miRNAs in body fluids.

The recent advances in the field of miRNA research, which include technical/technological advances and knowledge, are providing better conditions and tools for the study of circulating miRNAs. Examples of promising recent investigations regarding the potential diagnostic value of circulating miRNA signatures for lung cancer were described in this review. In addition, although there are very few in existence, two clinical trials may provide results for the potential clinical applicability of miRNA signatures in the near future. Therefore, expectations are still high regarding the potential clinical applicability of circulating miRNAs as biomarkers, which will probably lead to better planned strategies and the use of standardized methodology for upcoming work performed in the field.

We should also keep in mind that lung cancer is a multifactorial and highly heterogenous disease, and consequently, it is probable that multiple and combined strategies are required to achieve effective methods of diagnosis and follow-up that can be integrated into clinical practice.

Therefore, the future of the clinical applicability of miRNAs as non-invasive biomarkers probably lies in the use of miRNA signatures in combination with other methods of screening for lung cancer, such as LCDC and CT. Furthermore, diagnostic signatures of circulating miRNAs could be used to complement other types of markers that are currently being investigated for lung cancer diagnosis in tumor liquid biopsies, such as circulating tumor cells (CTCs) and circulating cell-free tumor DNA (ctDNA) specifically targeting mutated tumor-specific DNA [116,117].

## Figures and Tables

**Table 1:** Signatures of circulating miRNAs as potential diagnostic biomarkers of lung cancer in peripheral blood.

miRNA Signature	Sample Type	Type of Biomarker	Cohort Size	Method of Quantification and Normalization	References
**Signature #1**: miR-1285-3p, miR-205-5p, miR-1260a, miR-1260b, miR-3152-3p, miR-378b, miR-1202, miR-139-5p, miR-16-2-3p, miR-18a-3p, miR-23b-3p, miR-3907, miR-551b-3p, and miR-93-3p (LC vs. all other groups); **Signature #2**: miR-1285-3p miR-205-5p, miR-17-3p, miR-1202, let-7g-3p, miR-193a-5p, miR-21-3p, miR-3610, miR-4282, miR-4286, miR-452-3p, miR-516a-3p, miR-572, and miR-625-5p (LC vs. nontumor lung disease); **Signature #3**: miR-1285-3p, miR-205-5p, miR-1260a, miR-1260b, miR-3152-3p, miR-378b, miR-17-3p, miR-564, and miR-374b-5p (early-stage LC vs. without LC)	Whole blood	Diagnostic (LC symptomatic patients)	LC (*n* = 606); nontumor lung disease (*n* = 593); other diseases not affecting lungs (*n* = 883); and control subjects (*n* = 964)	Microarray; quantile normalization	Fehlmann et al., 2020 [85]
let-7a-5p, miR-1-3p, miR-1291, miR-214-3p, and miR-375	Serum	Diagnostic (early-stage NSCLC)	NSCLC stages I and II (*n* = 744) and matched controls (*n* = 944)	qPCR; a Absolute expression	Ying et al., 2020 [86]
miR-1268b and miR-6075	Serum	Diagnostic (resectable lung cancer)	LC (*n* = 1566) and noncancer controls (*n* = 2178)	Microarray; internal controls miR-4463, miR-2861, and miR-1493-p	Asakura et al., 2020 [87]
**Signature #1**: miR-17, miR-190b, miR-19a, miR-19b, miR-26b, and miR-375 (LC vs. healthy controls); **Signature #2**: miR-17, miR-190b, and miR-375 (NSCLC vs. SCLC)	Plasma	Diagnostic (LC vs. healthy controls; NSCLC vs. SCLC)	LC (*n* = 676; 533 NSCLC and 137 SCLC); high-risk healthy subjects (*n* = 456)	Microarray and qPCR; endogenous control miR 1228	Lu et al., 2018 [88]
**Signature #1**: miR-16-5p, miR-92a, miR-451a, miR-106b-5p, miR-155-5p, miR-217, miR-1285-3p, and miR-1285-5p; **Signature #2**: miR-16-5p, miR-148b-3p, miR-378e, miR-484, and miR-664a-3p; **Signature #3**: miR-16-5p, miR-92a, and miR-451a	Plasma	Diagnostic (early-stage NSCLC)	AD and SCC (*n* = 139) and nondisease controls (*n* = 61).	RNA sequencing and qPCR; global normalization and exogenous ath-miR-159 or endogenous miR-93 control	Reis et al., 2020 [83]
miR-33a-5p and miR-28-3p	Whole blood	Diagnostic	LC (*n* = 90) and healthy controls (*n* = 90); LC stages I–II (*n* = 41); healthy controls (*n* = 41)	qPCR; snRNA U6	Pan et al., 2018 [89]
Exosomal miRNAs **Signature#1**: miR-181b-5p, miR-361b-5p, miR-10b-5p, and miR-320b (NSCLC vs. healthy controls); **Signature#2**: miR-181-5p and miR-361-5p (AD vs. NSCLC); **Signature#3**: miR-320b and miR-10b-5p (SCC vs. NSCLC)	Plasma	Diagnostic (NSCLC vs. Healthy controls; AD vs. NSCLC; SCC vs. NSCLC)	NSCLC (*n* = 88; 57 AD and 31 SCC) and healthy controls (*n* = 42)	RNA sequencing and qPCR; RPM-mappable miRNA sequences and cel-miR-39	Jin et al., 2017 [81]
Exosomal miRNAs miR-5684 and miR-125b-5p	Serum	Diagnostic (NSCLC vs. healthy controls)	NSCLC (*n* = 330) and healthy controls (*n* = 312)	Microarrays and qPCR; miRNAs withintensities ≥30 and snRNA U6	Zhang et al., 2020 [90]
Exosomal miRNAs miR-20b-5p and miR-3187-5p	Serum	Diagnostic (NSCLC vs. healthy controls)	NSCLC (*n* = 276) and healthy controls (*n* = 282)	Microarrays and qPCR; miRNAs withintensities ≥30 and snRNA U6	Zhang et al., 2020 [91]

LC: lung cancer; NSCLC: non-small-cell lung cancer; AD: adenocarcinoma; SCC: squamous cell carcinoma; snRNA: small nuclear RNA; RPM: read count per million.

**Table 2:** Signatures of circulating miRNAs as potential diagnostic biomarkers of lung cancer in other body fluids.

miRNA Signature	Sample Type	Type of Biomarker	Cohort Size	Method of Quantification and Normalization	References
miRNA-126 and let-7a	Bronchoalveolar lavage (BAL)	Diagnostic (early-stage AD)	AD (*n* = 13) and nontumor pathology controls (*n* = 15)	qPCR; endogenous control miR-30a-5p	Kim et al., 2018 [67]
miR-1285, miR-1303, miR-29a-5p, and miR-650	Bronchial lavage (BL)	Diagnostic (LC)	LC (*n* = 30) and noncancer controls (*n* = 30)	qPCR; exogenous control cel-miR 39	Rehbein et al., 2015 [93]
miR-198 (combined with carcinoembryonic antigen (CEA) and cytokeratin 19 fragment (CYFRA 21-1))	Pleural effusion (PE)	Diagnostic (AD with malignant pleural effusion (MPE) vs. patients with benign pleural effusion (BPE))	MPE (*n* = 52) and BPE patients (*n* = 55)	Microarray and qPCR; endogenous miR-192 and snRNA U6	Han et al., 2013 [13]
miR-21(miRNA from EVs)	Pleural lavage	Diagnostic, association with positive cytology and pleural invasion (AD)	AD (*n* = 41)	Digital PCR	Watabe et al., 2020 [68]
EVs-miRNAs miR-1-3p, miR-144-5p, and miR-150-5p	Pleural lavage	Diagnostic (NSCLC vs. BPE patients)	AD and SCC (*n* = 21) and BPE patients (*n* = 25)	PCR array; not specified	Roman-Canal et al., 2019 [12]
miR-31 and miR-210 *	Sputum *	Diagnostic, to improve the specificity of computed tomography (CT) (LC vs. cancer-free smokers)	LC (*n* = 130) and cancer-free smokers (*n* = 141)	qPCR; snRNA U6	Shen et al., 2014 [11]

LC: lung cancer, AD: adenocarcinoma; MPE: malignant pleural effusion; BPE: benign pleural effusion; EVs: extracellular vesicles. * miRNAs are from the cell pellet collected from the sputum by centrifugation; therefore, they are endogenous miRNAs.

**Table 3:** Circulating miRNAs as potential therapy response biomarkers of lung cancer.

miRNA Signature	Sample Type	Type of Biomarker	Cohort Size	Method of Quantification and Normalization	References
199a-3p, miR-21-5p, and miR-28-5p	Plasma	Biomarker for anti-PD-1/PD-L1 antibody therapy response in advanced NSCLC	NSCLC (*n* = 50), 22 responders and 28 non-responders	Sequencing and qPCR; RPM of processed reads and endogenous miR-30a-5p	Shukuya et al., 2020 [96]
Exosomal miRNAs miR-320d, miR-320c, and miR-320b	Plasma	Biomarker for anti-PD-1/PD-L1 therapy response in EGFR/ALK-negative advanced NSCLC	NSCLC (*n* = 9), 5 responders and 4 non-responders	Sequencing; not specified	Peng et al., 2020 [97]
miR-93, miR-138- 5p, miR-200, miR-27a, miR-424, miR-34a, miR-28, miR-106b, miR-193a-3p, and miR-181a	Serum	Biomarker for anti-PD-1 therapy response in EGFR/ALK-negative advanced NSCLC	NSCLC, stage IV (*n* = 80), 36 responders and 44 non-responders	PCR array and qPCR; not specified and serum volume	Fan et al., 2020 [98]
miR-30b and miR-30c	Plasma	Biomarker for erlotinib response in EGFR-mutated NSCLC	EGFR-mutated NSCLC patients (*n* = 29)	qPCR; absolute expression	Hojbjerg et al., 2019 [99]

NSCLC: non-small-cell lung cancer; PD-1: programmed cell death protein 1; PD-L1: programmed cell death protein 1 ligand; RPM: read count per million; EGFR: epidermal growth factor receptor; ALK: anaplastic lymphoma receptor tyrosine kinase; EGFR: epidermal growth factor receptor.

**Table 4:** Circulating miRNAs as potential prognosis biomarkers of lung cancer.

miRNA Signature	Sample Type	Type of Biomarker	Cohort Size	Method of Quantification and Normalization	References
miR-590-5p	Plasma	Prognosis (NSCLC) Low levels: lower median survival rates	NSCLC (*n* = 80) and healthy controls (*n* = 80)	qPCR; Cel-miR-39	Khandelwal et al., 2020 [101]
**Signature #1**: miR-18a, miR-20a, miR-92a, miR-126, miR-210, and miR-19a; **Signature 2**: miR-18a, miR-20a, miR-92a, miR-210, and miR-126	Plasma	Prognosis (NSCLC) Signature #1: high levels, worse DFS Signature #2: high levels, sorter OS	NSCLC (*n* = 196)	qPCR; snRNA U6	Xu et al., 2018 [102]
miR-34a and miR-34c	Plasma	Prognosis (NSCLC) High levels miR-34a: prolonged DFS High levels miR-34c: longer OS	NSCLC (*n* = 196)	qPCR; snRNA U6	Zhao et al., 2017 [103]
miR-215-5p, miR-411-3p, miR-493-5p, miR-494-3p, miR-495-3p, miR-548j-5p, and miR-93-3p	Serum	Prognosis (NSCLC after nivolumab treatment) Signature associated with OS >6 months	NSCLC (*n* = 51)	Sequencing and qPCR; TMM normalization and endogenous miR-93-5p and miR-222-3p	Halvorsena et al., 2018 [104]

NSCLC: non-small-cell lung cancer; DFS: disease-free survival; OS: overall survival; TMM: trimmed mean of M-values.

**Table 5:** Clinical trials that investigated the diagnostic value of circulating miRNAs in lung cancer. Clinical trials registered at the US National Institutes of Health database (ClinicalTrials.gov) using the keywords “miRNA and lung cancer” (search results from 12 January 2021).

Title of Study	Status	Completion Date	Enrollment	Analysis	Study Results	Pre-Clinical References
Plasma microRNA Profiling as First Line Screening Test for Lung Cancer Detection: A Prospective Study	Active, not recruiting	September 2021	4119 participants (healthy heavy smokers)	Pre-selected panel of 24 miRNAs in plasma	Study not completed	[31,114,115]
Addition of microRNA Blood Test to Lung Cancer Screening Low Dose CT	Active, not recruiting	August 30, 2020	479 participants undergoing LDCT (smokers)	Hummingbird Diagnostic´s microRNA test for lung cancer diagnosis in total blood	No results available	Not provided
MicroRNA Genetic Signature in NSCLC Egyptian Patients	^†^ Completed	August 2016	40 participants (20 NSCLC, 10 nonsmokers, 10 smokers)	miRNA signature pattern in NSCLC Egyptian patients (by microarray)	^†^ No results available	Not provided
Quantification of microRNAs in Diagnosis of Pulmonary Nodules	Completed	May 2019	103 participants (patients with nodules >5 mm and <30 mm in the lung parenchyma)	Pre-selected panel of 34 miRNAs	No results available	Not provided
Plasma miRNAs Predict Radiosensitivity of Different Fractionation Regimes in Palliative Radiotherapy for Advanced Non-small Cell Lung Cancer: Multicenter Controlled Study (RadimiR-01)	^†^ Unknown	Last update posted: March 2017	Estimated 240 participants (NSCLC)	Unspecified miRNAs in plasma	^†^ No results available	Not provided
Association between VEGF-C and miRNA and Clinical Non-small Cell Lung Cancer and Esophagus Squamous Cell Carcinoma	^†^ Unknown	Last update posted: Nov 2010	Estimated 250 participants (NSCLC)	Unspecified miRNAs in unspecifies biological samples, probably serum	^†^ No results available	Not provided

LDCT: low-dose computed tomography; NSCLC: non-small-cell lung cancer; ^†^ Study has passed its completion date, and status has not been verified in more than two years.

## Data Availability

No new data were created or analyzed in this study. Data sharing is not applicable to this article.
